# Micronodular T-cell/histiocyte-rich B-cell lymphoma of the spleen in a case of small lymphocytic lymphoma: A Richter's transformation

**DOI:** 10.3109/03009731003686920

**Published:** 2010-07-19

**Authors:** Hüseyin Kemal Türköz, Nedim Polat, Ilker Akin, Deniz Özcan

**Affiliations:** ^1^Department of Pathology, Marmara University School of Medicine, Uskudar, IstanbulTurkey; ^2^Department of Pathology, Sisli Etfal Education and Research Hospital, IstanbulTurkey; ^3^Department of Pathology, Okmeydani Education and Research Hospital, IstanbulTurkey

**Keywords:** Large B-cell lymphoma, spleen, T-lymphocytes

## Abstract

A case of micronodular T-cell/histiocyte-rich B-cell lymphoma of the spleen who had a prior diagnosis of small lymphocytic lymphoma is presented. Micronodular T-cell/histiocyte-rich B-cell lymphoma of the spleen was first described in 2003, and very few cases have been reported since then. This is the first reported case supervening in a patient with pre-existing chronic lymphocytic lymphoma. We review its clinical, pathologic, and immunohistochemical features and the difficulties we encountered during diagnosis.

## Introduction

A new variant of T-cell/histiocyte-rich large B-cell lymphoma in the spleen was first described by Dogan et al. in 2003 (1). This new variant was reported to show a micronodular pattern and no macroscopically identifiable tumor mass in the spleen. Tumor cells consisted of large, atypical B-cells scattered in T-cell and histiocyte-rich micronodules. Only a few cases have been reported since Dogan et al. described this new lymphoma subtype (2–5). We report here a case of micronodular T-cell/histiocyte-rich large B-cell lymphoma of the spleen that was diagnosed with chronic lymphocytic lymphoma 9 years ago.

## Case report

**Figure 1.**
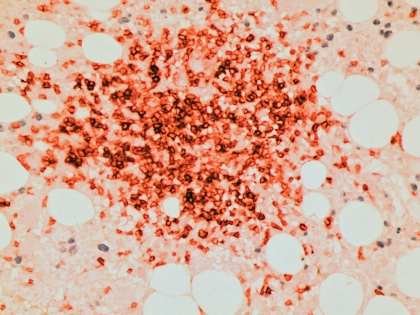
Most of the cells in the lymphoid aggregates were CD3(+) small T-lymphocytes (trephine biopsy, CD3 antibody, counterstained with hematoxylin, 400×).

**Figure 2.**
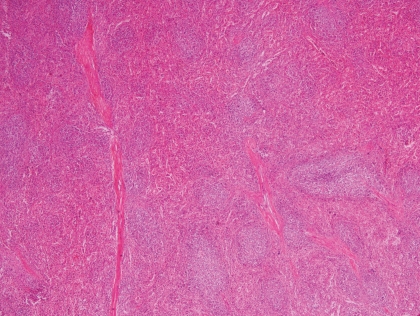
White pulp was replaced by micronodular lymphoid aggregates in the spleen (hematoxylin-eosin, 40×).

**Figure 3.**
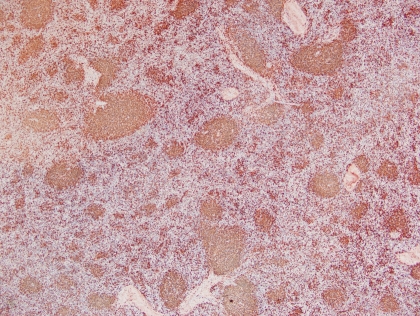
A total of 90%–95% of the cells in the micronodules were CD3(+) small T-cells (spleen, CD3 antibody, counterstained with hematoxylin, 40×).

**Figure 4.**
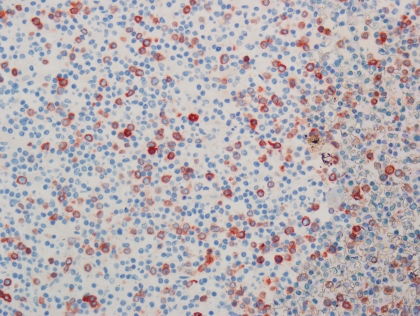
Scattered large centroblastic cells in the micronodules were CD20(+) B-cells (spleen, CD20 antibody, counterstained with hematoxylin, 400×).

A 57-year-old man was suffering recently from fatigue, dyspnea, night sweats, and weight loss. He had a history of chronic lymphocytic leukemia/small lymphocytic lymphoma and was treated with cyclophosphamide, vincristine, and prednisone in 2000. He was in remission for the last 9 years. Physical examination revealed pallor and splenomegaly. There was no sign of peripheral lymphadenopathy. Routine blood count disclosed pancytopenia. Abdominal ultrasonography revealed splenomegaly. Computed tomography confirmed that splenomegaly was isolated and there was no accompanying lymphadenopathy. Clinical diagnosis was relapse of small lymphocytic lymphoma. A bone-marrow biopsy was performed for pathologic examination. Histological examination of the trephine biopsy showed lymphoid aggregates with an intertrabecular pattern. There were a few large lymphocytes with vesicular nuclei. Immunohistochemical study revealed that 90% of the lymphoid cells were CD3 (+) small T-cells and approximately 10% of the population was CD20(+) B-cells (Figure 1). There was no reactivity with bcl-1, bcl-2, CD5, CD23, and CD10 antibodies. These histological data, especially predominance of T-cells, suggested that the infiltration was reactive and was not consistent with a relapse of patient's previous small lymphocytic lymphoma. Following bone-marrow examination, a diagnostic and therapeutic splenectomy has been undertaken. The spleen measured 36 cm × 21 cm × 13 cm. The cut surface showed white nodules with diameters from 1 to 3 mm within the normal-appearing red pulp. Microscopic examination showed that these nodules consisted of small to medium-sized lymphocytes and histiocytes. Most of the nodules also contained scattered large lymphoid cells with large vesicular nuclei and two to three nucleoli. These micronodules replaced all white pulp, and there was no remaining uninvolved white pulp (Figure 2). Immunohistochemical study showed that small cells accounting for 90%–95% of the nodules were CD3(+) T-cells (Figure 3). Scattered large centroblastic cells were positive for CD20, bcl-6, and CD79a (Figure 4) but negative for CD5, bcl-2, CD10, CD30, CD15, epithelial membrane antigen (EMA), CD23, bcl-1, and Epstein-Barr virus/latent membrane proteins. Ki-67 proliferation index for centroblastic cells was around 90%.

## Discussion

Micronodular T-cell/histiocyte-rich B-cell lymphoma of the spleen was first reported and described by Dogan et al. in 2003 (1). A few more cases with the same features have been reported since then (2–5). All the reported cases of micronodular T-cell/histiocyte-rich B-cell lymphoma of the spleen presented with splenomegaly, anemia, and B symptoms. There was no or minimal lymphadenopathy, which was usually limited to hilar lymph nodes of the spleen.

Micronodular T-cell/histiocyte-rich B-cell lymphoma of the spleen involves white pulp in a micronodular pattern. Microscopically, there are scattered large cells in a background of CD3(+) T-cells and CD68(+) histiocytes. The large tumor cells express B-cell markers CD20 and bcl-6. They are variably positive for EMA, bcl-2, and CD30 and negative for CD10 and Epstein-Barr virus/latent membrane proteins. Since no follicular dendritic cell mesh-work can be demonstrated by CD21, Dogan et al. advised this variant of lymphoma to be classified and managed as a variant of diffuse large B-cell lymphoma (1).

All morphological and immunohistochemical features in our patient are consistent with the diagnosis of micronodular T-cell/histiocyte-rich B-cell lymphoma of the spleen.

Dogan et al. reported that diagnosis of micronodular T-cell/histiocyte-rich B-cell lymphoma of the spleen was very difficult if the marrow biopsy was examined without the knowledge of the splenic histology, as was the case for our patient (1). They informed that features in bone-marrow might suggest follicular lymphoma, B-chronic lymphocytic leukemia, mantle cell lymphoma, classical Hodgkin's lymphoma, nodular lymphocyte predominant Hodgkin's lymphoma, T-cell lymphoma, and reactive changes, and a precise diagnosis might be very difficult without a splenectomy. Our patient's history of small lymphocytic lymphoma further complicated an accurate diagnosis. Bone-marrow biopsy revealed a T-cell-rich infiltrate, and there were no histologic findings consistent with small lymphocytic lymphoma. Prior history of small lymphocytic lymphoma in our case led us to a probable diagnosis of reactive changes in bone-marrow biopsy and a splenectomy was needed for accurate diagnosis.

Apart from Wang et al., no reported cases were associated with a different lymphoma type. Wang et al. reported co-occurrence of splenic marginal zone lymphoma and micronodular T-cell/histiocyte-rich large B-cell lymphoma in the spleen. Similar to other indolent lymphoid neoplasms, splenic marginal zone lymphoma may undergo transformation to large B-cell lymphoma (6). Wang et al. speculated that their case could be a splenic marginal zone lymphoma undergoing transformation to micronodular T-cell/histiocyte-rich B-cell lymphoma of the spleen (4).

Our patient had a history of small lymphocytic lymphoma. Small lymphocytic lymphoma/chronic lymphocytic leukemia is characterized by a variable clinical course. A subset of patients has indolent disease for decades, whereas others develop a secondary aggressive lymphoma, mostly diffuse large B-cell lymphoma. This event has been termed Richter's transformation (7). The interval between the initial diagnosis and Richter's transformation ranges from 0 to 10 years (8). Micronodular T-cell/histiocyte-rich B-cell lymphoma of the spleen is an aggressive neoplasm and was advised to be managed as a variant of diffuse large B-cell lymphoma (1). Micronodular T-cell/histiocyte-rich B-cell lymphoma of the spleen in our case seems to represent a Richter's syndrome.

Diagnosis of micronodular T-cell/histiocyte-rich B-cell lymphoma of the spleen in a bone-marrow biopsy could be very difficult. Follicular lymphoma, B-chronic lymphocytic leukemia, mantle cell lymphoma, Hodgkin's lymphoma, T-cell lymphomas, and especially reactive changes should be counted as differential diagnoses. It is an aggressive neoplasm and can be transformed from an indolent lymphoma. Additional reports and studies are needed to establish features of this rare B-cell lymphoma variant.

## References

[1] Dogan A, Burke JS, Goteri G, Stitson RN, Wotherspoon AC, Isaacson PG (2003). Micronodular T-cell/histiocyte-rich large B-cell lymphoma of the spleen: histology, immunophenotype, and differential diagnosis. Am J Surg Pathol.

[2] Mollejo M, Algara P, Mateo MS, Menárguez J, Pascual E, Fresno MF (2003). Large B-cell lymphoma presenting in the spleen: identification of different clinicopathological conditions. Am J Surg Pathol.

[3] Li S, Mann KP, Holden JT (2004). T-cell-rich B-cell lymphoma presenting in the spleen: a clinicopathological analysis of 3 cases. Int J Surg Pathol.

[4] Wang SA, Olson N, Zukerberg L, Harris NL (2006). Splenic marginal zone lymphoma with micronodular T-cell-rich B-cell lymphoma. Am J Surg Pathol.

[5] Kan E, Levy I, Benharroch D (2008). Splenic micronodular T-cell/histiocyte-rich large B-cell lymphoma. Ann Diagn Pathol.

[6] Camacho FI, Mollejo M, Mateo MS, Algara P, Navas C, Hernández JM (2001). Progression to large B-cell lymphoma in splenic marginal zone lymphoma: a description of a series of 12 cases. Am J Surg Pathol.

[7] Lortholary P, Ripault M, Boiron M (1964). [Richter's Syndrome]. Nouv Rev Fr Hematol.

[8] Harousseau JL, Flandrin G, Tricot G, Brouet JC, Seligmann M, Bernard J (1981). Malignant lymphoma supervening in chronic lymphocytic leukemia and related disorders. Richter's syndrome: a study of 25 cases. Cancer.

